# Where are the alcohol advertising hotspots near schools?

**DOI:** 10.1002/hpja.896

**Published:** 2024-06-26

**Authors:** Joelie Mandzufas, Karen Lombardi, Robyn S. Johnston, Alexia Bivoltsis, Justine Howard, Paula Hooper, Gina S. A. Trapp

**Affiliations:** ^1^ Telethon Kids Institute West Perth Australia; ^2^ The University of Western Australia Perth Australia; ^3^ Edith Cowan University Joondalup Australia; ^4^ Present address: Nutrition & Health Innovation Research Institute, School of Medical and Health Sciences Edith Cowan University Joondalup Australia

**Keywords:** alcohol, children, food advertising, INFORMAS, marketing, public health, schools

## Abstract

**Issue Addressed:**

Physically locating liquor stores near schools can strongly influence the chances of youth accessing and consuming alcohol, and may also increase children's exposure to alcohol advertising. Investigating the association between the presence of a liquor store near a school and the prevalence of outdoor alcohol advertising is crucial from a policy perspective, as it can inform future regulations on the placement of liquor stores and outdoor advertising near educational institutions.

**Methods:**

All outdoor alcohol advertising within a 500 m radius (audit zone) of 64 randomly selected schools from local government areas across metropolitan Perth was identified by direct observation; recording the size, setting, location and content of each advertisement. Results were compared based on whether the school audit zone contained a liquor store or not.

**Results:**

Over half (*n* = 36, 56%) of all school audit zones had at least one alcohol advertisement, with an average number of 5.9 alcohol advertisements per zone (SD = 10.2). The majority (97.9%) of advertisements were in the 38 audit zones containing a liquor store (average = 9.7, SD = 11.9 per zone), compared to zones without a liquor store (average = .3, SD = .7 per zone).

**Conclusions:**

Perth school zones containing a liquor store in their 500 m radius had, on average, 30 times more outdoor alcohol advertising, compared with school zones without a nearby liquor store.

**So What?:**

The siting of liquor stores and the display of alcohol advertisements around educational settings require combined policy, planning and public health approaches to mitigate children's exposure to alcohol marketing, especially during school transit.

## 1 INTRODUCTION

The Australian Guidelines to Reduce Health Risks from Drinking Alcohol recommend that children under the age of 18 do not drink any alcohol.^1^ Indeed, the younger the age of initiation to alcohol consumption, the greater the risks of harm.^2^ Young people's attitudes towards alcohol, intentions to consume, and alcohol consumption behaviours are significantly shaped by their exposure to various forms of alcohol marketing.^3^, ^4^, ^5^ Alcohol marketing includes a wide range of advertising channels such as outdoor advertising, digital media, print ads and promotional events. The strategic placement and content of alcohol advertisements are often designed to appeal to the younger demographic, and these marketing efforts often portray alcohol consumption in a positive light, which can subtly influence the perceptions and attitudes of young individuals towards alcohol.^6^ Thus, reducing children's exposure to alcohol advertising is an important policy consideration.^7^

Outdoor advertising has been shown to be a significant source of alcohol advertising exposure for school‐aged children,^8^ with opportunities for frequent and repetitive viewing during students' transit to and from school.^9^ Whilst a 2019 audit of bus shelters located near Perth schools found only a relatively low number of alcohol advertisements, the authors concluded that ‘given the harms associated with alcohol consumption among young people, the placement of any alcohol advertising near schools is inconsistent with policy recommendations from leading health and medical organisations’.^10^ An audit of outdoor advertising in close proximity to schools in three regions in Spain revealed a high proportion of advertisements for alcohol products.^11^ Similar research conducted in Uganda found almost a quarter of branded advertisements around 25 schools were for alcohol.^12^ It has been established that the presence of a liquor store near school at age 14 years is a significant predictor of alcohol consumption at age 17 years.^13^ It is reasonable to expect an increased density of alcohol advertising where liquor stores are present, yet to date only limited research has linked the presence of liquor stores near schools with children's exposure to advertising. A US study found that most alcohol advertisements in school audit zones were proximal to liquor stores or licensed outlets.^4^ Furthermore, a New Zealand study involving wearable cameras found a strong association between the number of liquor stores within 500 m of a school and children's recorded exposure to alcohol advertising.^14^ It is important to understand if these findings are supported in differing regulatory and cultural contexts. Therefore, the aim of this study was to explore the association between the presence of a liquor store near school and the prevalence of alcohol outdoor advertising in Perth, Western Australia. The findings will provide valuable insights into the local Australian context, informing evidence‐based policy, planning and public health interventions to reduce young people's exposure to alcohol advertising.

## 2 METHODS

These data were drawn from an audit of all outdoor advertising in selected school audit zones in Perth, Western Australia. The outdoor advertising audit methodology has been described in detail elsewhere (see^9^) and was based on ‘INFORMAS protocol: Outdoor advertising (school zones)’,^15^ with data collection occurring July–December 2019. Ethics exemption was granted from The University of Western Australia Human Research Ethics Committee (Ref RA/4/20/4714).

### 2.1 School selection

Australian Local Government Areas (LGAs) are districts of varying size that manage their local affairs. Following the INFORMAS ‘Optimal’ sampling approach to gain a representative sample of locality and socio‐economic status, the 38 LGAs in the Greater Perth metropolitan area were split into low and high socio‐economic area groupings, measured by the Australian Bureau of Statistics Socio‐Economic Index for Areas (SEIFA) Index of Relative Socio‐Economic Disadvantage.^15^ Eight low socio‐economic LGAs and eight high socio‐economic LGAs were then randomly selected, with four schools randomly sampled from each LGA for inclusion, totalling 64 schools: 33 schools with only primary school students (up to Year 6), and 31 schools with some secondary students within the school.

### 2.2 Data collection

Maps were created in ArcGIS showing the streets contained within a 500 m radial buffer from each school boundary (audit zone). Research assistants were trained in the use of the data collection instrument and the definitions of advertisements and their characteristics. Each research assistant completed a data collection in a test zone, with data compared to a gold standard collected by a senior researcher. Following this comparison, further training was provided if required. Pairs of research assistants walked every street on the navigation map for all 64 school zones and photographed all outdoor advertising visible from the street.^9^ Audits were conducted during weekdays, during times that students would be in transit or at school, to ensure that moveable advertisements visible to students would be in place. An outdoor advertisement was defined as any signage or advertisement for a product, brand or service located outdoors or on external walls or windows of buildings. Signage was excluded if its primary purpose was store identification only (e.g., the name or logo of the business and phone number or opening hours) with no other promotional aspect. Tablet devices were used to record information about the size (small, medium, large), setting (food or non‐food shop, roadside, bus shelter, building) and type (poster or banner, free‐standing, merchandising, billboard, painted wall or digital sign) of outdoor advertisement, with the location (latitude and longitude) automatically recorded. The information recorded for each advertisement was agreed by two field researchers to ensure accuracy, allowing for intra‐ and inter‐zone comparisons. For this study, senior researchers created a subset of advertisements originally coded as alcohol and examined photographs and locations to conduct checks of the field coding and to further categorise food shops into food outlets (where alcohol is advertised with a meal), liquor stores (where alcohol can be purchased for consumption elsewhere), and bars or nightclubs (where alcohol can be purchased for consumption on the premises). For this analysis, school audit zones were categorised as containing at least one liquor store, or no liquor stores. The presence or absence of liquor stores within the audit zone was initially determined via a visual search of businesses within the audit zones on Google maps and then validated by ground‐truthing, with full agreement between these methods.

### 2.3 Statistical analysis

All data were analysed using R and R Studio.^16^ Descriptive statistics were generated to describe the frequency and proportion of alcohol advertisements within audit zones stratified by presence of a liquor store. The Welch two‐sample *t*‐test was used to compare the average number of advertisements between zones with and without a liquor store.

## 3 RESULTS

Table 1 presents the count and proportion of outdoor alcohol advertisements within a 500 m radius of Perth schools, stratified by the presence of a liquor store. Across all 64 school audit zones combined, a total of 376 outdoor advertisements for alcohol were identified. Over half (*n* = 36, 56%) of the school audit zones had at least one alcohol advertisement, with the average number of alcohol advertisements per zone being 5.9 (SD = 10.2). School audit zones containing a liquor store (*n* = 38, 59%) had a total of 368 outdoor alcohol advertisements (average = 9.7, SD = 11.9 per school audit zone), and zones were most likely to have between one and five (34.2%) or between 11 and 20 (31.6%) advertisements for alcohol. These differences in frequency between zones with and without liquor stores were significant (*p* < .001). School audit zones without a liquor store (*n* = 26, 41%), had a total of eight outdoor alcohol advertisements (average = .3, SD = .7 per school audit zone), and no school zone had more than five advertisements for alcohol. School audit zones located in higher socio‐economic areas (*n* = 32) had a total of 284 outdoor alcohol advertisements (average = 8.9, SD = 13.2 per school audit zone), compared to those zones in lower socio‐economic areas (*n* = 32) with 92 advertisements (average = 2.9, SD = 4.5 per school audit zone). In the 33 audit zones of schools serving only primary school students, there were 182 outdoor alcohol advertisements (average = 5.5, SD = 7.4). Audit zones of schools with secondary students had a total of 194 outdoor alcohol advertisements (average = 6.3, SD = 12.7). The majority (63%) of all the alcohol advertisements identified were located on/outside a liquor outlet (liquor store or bar/nightclub) as opposed to on/outside other food businesses (2%), along the roadside (30.9%), on/outside buildings (.3%), on a bus shelter (3%) or non‐food business (.5%).

**TABLE 1:** Count and proportion of outdoor alcohol advertisements within a 500 m radius of Perth schools stratified by presence of a liquor store.

	All school audit zones (*n* = 64)	School audit zones with a liquor store present within a 500 m radius (*n* = 38)	School audit zones without a liquor store present within a 500 m radius (*n* = 26)	*p*‐Value^a^
	*N* (%)	*N* (%)	*N* (%)	
Total outdoor alcohol advertisements	376	368	8	
Average outdoor alcohol advertisements per zone (SD)	5.9 (10.2)	9.7 (11.9)	.3 (.7)	<.001***
Frequency of outdoor alcohol advertisements in school audit zones				
NIL	28 (43.8%)	7 (18.4%)	21 (80.8%)	
1 to 5 advertisements	18 (28.1%)	13 (34.2%)	5 (19.2%)	
6 to 10 advertisements	2 (3.1%)	2 (5.3%)	0	
11 to 20 advertisements	12 (18.8%)	12 (31.6%)	0	
21 to 50 advertisements	3 (4.7%)	3 (7.9%)	0	
More than 50 advertisements	1 (1.6%)	1 (2.6%)	0	
Audit zones and advertisements by area‐level socio‐economic level (SES)				
High SES school audit zones				
Number of school audit zones	32	23	9	
Total outdoor alcohol advertisements	284	284	0	
Average number of outdoor alcohol advertisements per school audit zone (SD)	8.9 (13.2)	12.3 (5.6)	0 (0)	<.001***
Low SES school audit zones				
Number of school audit zones	32	15	17	
Total outdoor alcohol advertisements	92	84	8	
Average number of outdoor alcohol advertisements per school audit zone (SD)	2.9 (4.5)	14.1 (5.5)	.5 (.9)	<.001***
Audit zones and advertisements by school type				
Schools serving primary students only				
Number of school audit zones	33	20	13	
Total outdoor alcohol advertisements	182	178	4	
Average number of outdoor alcohol advertisements per school audit zone (SD)	5.5 (7.4)	8.9 (7.9)	.3 (.6)	<.001***
Schools with secondary students				
Number of school audit zones	31	18	13	
Total outdoor alcohol advertisements	194	190	4	
Average number of outdoor alcohol advertisements per school audit zone (SD)	6.3 (12.7)	10.6 (15.4)	.3 (.9)	<.001***
Size of outdoor alcohol advertisements				
Small (>A4 size, but <1.3 × 1.9 m)	325 (86.4%)	319 (86.7%)	6 (75.0%)	
Medium (>1.3 × 1.9 m but <2.0 × 2.5 m)	37 (9.8%)	36 (9.8%)	1 (12.5%)	
Large (≥2 × 2.5 m)	14 (3.7%)	13 (3.5%)	1 (12.5%)	
Type of alcohol advertisements				
Billboard	3 (.8%)	2 (.5%)	1 (12.5%)	
Poster or banner	246 (65.4%)	241 (65.5%)	5 (62.5%)	
Free‐standing	117 (31.1%)	115 (31.3%)	2 (25.0%)	
Painted building or wall	2 (.5%)	2 (.5%)	0 (.0%)	
Digital or LED sign	7 (1.9%)	7 (1.9%)	0 (.0%)	
Merchandising	1 (.3%)	1 (.3%)	0 (.0%)	
Setting of alcohol advertisements				
Liquor store	218 (58.0%)	218 (59.2%)	0 (0%)	
Bar/nightclub	20 (5.3%)	20 (5.4%)	0 (0%)	
Food outlet	8 (2.1%)	5 (1.4%)	3 (37.5%)	
Roadside	116 (30.9%)	113 (30.7%)	3 (37.5%)	
Building	1 (.3%)	0 (0%)	1 (12.5%)	
Bus shelter	11 (2.9%)	11 (3.0%)	0 (0%)	
Non‐food business	2 (.5%)	1 (.3%)	1 (12.5%)	

*Note*: Bold indicates significant values *p* < 0.001.

^a^ Welch two sample *t*‐test used to compare averages in zones with and without a liquor store.

## 4 DISCUSSION

This study identified that over half of the Perth, Western Australia schools audited had outdoor alcohol advertising present within 500 m. Importantly, it was observed that the quantity of outdoor alcohol advertisements in the vicinity of schools was (on average) 30 times greater when a liquor store was present, and that zones without a liquor store had very few advertisements. Prior research focusing on the association between the proximity of liquor stores to schools and the extent of children's exposure to advertising is limited, however our results support their findings.^4^, ^14^

The presence of a liquor store in school audit zones poses significant concerns regarding the exposure of underage populations to outdoor alcohol advertising. Whilst the majority of advertisements were ‘small’ in this study, only advertisements that were at least A4 size were counted, and this category includes advertisements with dimensions up to 1.3 × 1.9 m. Advertisements of at least this size, and particularly where groups of advertisements are located together, provide opportunities for exposure to children in transit to and from school, no matter the mode of transport. Indeed, there is recent evidence of a dose–response relationship between exposure to alcohol advertising and drinking behaviours in adolescents.^5^ Our findings lend weight to the urgent need for policy changes to shape an environment that will reduce children's exposure to alcohol advertising, with the potential to reduce future impacts of underage drinking. Policy changes to be considered should include the revision of advertising guidelines or zoning laws to limit such advertisements near educational institutions, such as restrictions implemented in Ireland^17^ and Victoria.^18^ As the majority of alcohol advertisements in this study were located on or within 1 m of liquor outlets, any restrictions must address the proximity of liquor outlets to schools, which underscores the importance of thoughtful community planning and urban design. Given the complexities of state and local legislation, advocating for state‐level legislative support to provide a more robust framework for local decision‐making could be pivotal. This might include lobbying for state amendments that grant local governments greater autonomy in the placement and licensing of alcohol‐selling businesses based on public health considerations.

To complement policy change, the findings highlight the need for targeted public health messaging and educational programs in areas with high visibility of alcohol advertising. Health departments could use this information to develop localised strategies that counter the high levels of exposure to alcohol advertising, raising awareness about the risks of underage drinking and promoting healthy behaviours among young Australians.

In addition, community activism will be pivotal in advocating for changes to minimise children's exposure to alcohol advertising. As well as the role of the school curriculum in educating students about the impact of alcohol advertising, school communities are well placed to apply pressure for restrictions on potential exposures. Moreover, per the Children's Rights agenda, children have a right to be consulted, heard and to appropriately influence their environments. Thus, it is also important that future studies elicit schoolchildren's opinions on the prolific outdoor advertising of unhealthy foods and products near schools, so the voices of children are incorporated into the development of future policies. This is essential to prevent the design of purely adult environments that ignore the needs and perspectives of vulnerable population groups, such as children.^19^

A strength of this study was its representative sample of school audit areas, covering almost one half of all local government areas in the Perth metropolitan area, and inclusive of a range of socio‐economic areas and school types. The local contextual evidence produced by this study can inform targeted public health interventions such as community awareness campaigns or school‐based education programs in areas of high alcohol advertising exposure. However, this study was limited to one Australian city, with no regional data, and data were collected cross‐sectionally in 2019 with no recent data collected. Future research should investigate the awareness and actual exposure of alcohol advertising to young people, and the association of outdoor advertising with young people's alcohol consumption and monitor the effectiveness of any interventions implemented.

## 5 CONCLUSION

By demonstrating the relationship between the presence of liquor stores and prevalence of alcohol outdoor advertisements, this study adds to the global evidence base required to underpin policy change. Children's exposure to alcohol advertising could be reduced by regulating the location of liquor stores and controlling the propagation of alcohol promotions in areas nearby schools.

## FUNDING INFORMATION

This research was funded by Healthway and the Cancer Council Western Australia as part of their Rapid Obesity Policy Translation program. GT was supported by an Australian Research Council (DE210101791).

## CONFLICT OF INTEREST STATEMENT

The authors declare no conflicts of interest.

## ETHICS STATEMENT

Ethics Exemption Approval to conduct the study was given by the University of Western Australia Human Research Ethics Committee (Ref RA/4/20/4714).

## Data Availability

The data that support the findings of this study are available from the corresponding author upon reasonable request.

## References

[1] National Health and Medical Research Council . Australian guidelines to reduce health risks from drinking alcohol. Canberra: Commonwealth of Australia; 2020.

[2] Gardner LA , Stockings E , Champion KE , Mather M , Newton NC . Alcohol initiation before age 15 predicts earlier hazardous drinking: a survival analysis of a 7‐year prospective longitudinal cohort of Australian adolescents. Addiction. 2023;119(3):518–529. 37926434 10.1111/add.16376

[3] Sargent JD , Babor TF . The relationship between exposure to alcohol marketing and underage drinking is causal. J Stud Alcohol Drugs. 2020;19(2):113–124. 10.15288/jsads.2020.s19.113 PMC7063998 32079567

[4] Pasch KE , Komro KA , Perry CL , Hearst MO , Farbakhsh K . Outdoor alcohol advertising near schools: what does it advertise and how is it related to intentions and use of alcohol among young adolescents? J Stud Alcohol Drugs. 2007;68(4):587–596. 17568965 10.15288/jsad.2007.68.587

[5] Yoshida K , Kanda H , Hisamatsu T , Kuwabara Y , Kinjo A , Yoshimoto H , et al. Association and dose‐response relationship between exposure to alcohol advertising media and current drinking: a nationwide cross‐sectional study of Japanese adolescents. Environ Health Prev Med. 2023;28:58. 37766544 10.1265/ehpm.23-00127 PMC10569966

[6] Wells G , Trapp G , Wickens N , Heritage B . Powerful promotions: an investigation of the teen‐directed marketing power of outdoor food advertisements located near schools in Australia. Health Promot J Austr. 2024;35(1):144–153. 37012612 10.1002/hpja.724

[7] Burton RP , Henn CM , Lavoie DMA , O'Connor RBA , Perkins CM , Sweeney KB , et al. A rapid evidence review of the effectiveness and cost‐effectiveness of alcohol control policies: an English perspective. The Lancet (British Edition). 2017;389(10078):1558–1580. 10.1016/S0140-6736(16)32420-5 27919442

[8] Collins RL , Martino SC , Kovalchik SA , Becker KM , Shadel WG , D'Amico EJ . Alcohol advertising exposure among middle school‐age youth: an assessment across all media and venues. J Stud Alcohol Drugs. 2016;77(3):384–392. 27172570 10.15288/jsad.2016.77.384 PMC4869896

[9] Trapp G , Hooper P , Thornton L , Kennington K , Sartori A , Wickens N , et al. Children's exposure to outdoor food advertising near primary and secondary schools in Australia. Health Promot J Austr. 2021;33:642–648. 34418222 10.1002/hpja.532

[10] Parnell A , Edmunds M , Pierce H , Stoneham MJ . The volume and type of unhealthy bus shelter advertising around schools in Perth, Western Australia: results from an explorative study. Health Promot J Austr. 2019;30(1):88–93. 29577507 10.1002/hpja.55

[11] Fernandez‐Alvarez MM , Zabaleta‐del‐Olmo E , Cachero‐Rodríguez J , Martin‐Payo R . Nutritional content and quality of processed foods and beverages advertised near schools in three cities in the north of Spain. Nutr Bull. 2023;48(1):66–73. 36377713 10.1111/nbu.12597

[12] Dia OEW , Lovhaug AL , Rukundo PM , Torheim LE . Mapping of outdoor food and beverage advertising around primary and secondary schools in Kampala city, Uganda. BMC Public Health. 2021;21(1):707. 33845809 10.1186/s12889-021-10661-8 PMC8042698

[13] Trapp GSA , Knuiman M , Hooper P , Foster S . Proximity to liquor stores and adolescent alcohol intake: a prospective study. Am J Prev Med. 2018;54(6):825–830. 29656918 10.1016/j.amepre.2018.01.043

[14] Chambers T , Pearson AL , Kawachi I , Stanley J , Smith M , Barr M , et al. Children's home and school neighbourhood exposure to alcohol marketing: using wearable camera and GPS data to directly examine the link between retailer availability and visual exposure to marketing. Health Place. 2018;54:102–109. 30253378 10.1016/j.healthplace.2018.09.012

[15] Mackay S , Molloy J , Vandevijvere S . INFORMAS protocol: promotion outdoor advertising (School zones). 2017.

[16] R Core Team . R: a language and environment for statistical computing. Vienna, Austria: R Foundation for Statistical Computing; 2022.

[17] Government of Ireland . Public Health (Alcohol) Act 2018.

[18] Liquor Control Victoria . Responsible alcohol advertising and promotion guidelines. Richmond, Victoria: Victoria State Government; 2024.

[19] Christian H , Fried L , Dhamrait G , Nathan A , Beck B , Boruff B , et al. Built environments and child health: a policy review SSRN. 2021.

